# Fingerprinting the Substrate Specificity of M1 and M17 Aminopeptidases of Human Malaria, *Plasmodium falciparum*

**DOI:** 10.1371/journal.pone.0031938

**Published:** 2012-02-16

**Authors:** Marcin Poreba, Sheena McGowan, Tina S. Skinner-Adams, Katharine R. Trenholme, Donald L. Gardiner, James C. Whisstock, Joyce To, Guy S. Salvesen, John P. Dalton, Marcin Drag

**Affiliations:** 1 Division of Medicinal Chemistry and Microbiology, Faculty of Chemistry, Wroclaw University of Technology, Wroclaw, Poland; 2 Department of Biochemistry and Molecular Biology and Australian Research Council Centre of Excellence in Structural and Functional Microbial Genomics, Monash University, Clayton, Victoria, Australia; 3 Department of Biochemistry and Molecular Biology, Monash University, Clayton, Victoria, Australia; 4 Malaria Biology Laboratory, Queensland Institute of Medical Research, Brisbane, Queensland, Australia; 5 Griffith Medical Research College, Joint Program of Griffith University and the Queensland Institute of Medical Research, Brisbane, Queensland, Australia; 6 School of Medicine, The University of Queensland, Royal Brisbane Hospital, Brisbane, Queensland, Australia; 7 Institute of Parasitology, McGill University, Sainte Anne de Bellevue, Quebec, Canada; 8 Program in Apoptosis and Cell Death Research, Sanford Burnham Medical Research Institute, La Jolla, California, United States of America; Johns Hopkins Bloomberg School of Public Health, United States of America

## Abstract

**Background:**

*Plasmodium falciparum*, the causative agent of human malaria, expresses two aminopeptidases, *Pf*M1AAP and *Pf*M17LAP, critical to generating a free amino acid pool used by the intraerythrocytic stage of the parasite for proteins synthesis, growth and development. These exopeptidases are potential targets for the development of a new class of anti-malaria drugs.

**Methodology/Principal Findings:**

To define the substrate specificity of recombinant forms of these two malaria aminopeptidases we used a new library consisting of 61 fluorogenic substrates derived both from natural and unnatural amino acids. We obtained a detailed substrate fingerprint for recombinant forms of the enzymes revealing that *Pf*M1AAP exhibits a very broad substrate tolerance, capable of efficiently hydrolyzing neutral and basic amino acids, while *Pf*M17LAP has narrower substrate specificity and preferentially cleaves bulky, hydrophobic amino acids. The substrate library was also exploited to profile the activity of the native aminopeptidases in soluble cell lysates of *P. falciparum* malaria.

**Conclusions/Significance:**

This data showed that *Pf*M1AAP and *Pf*M17LAP are responsible for majority of the aminopeptidase activity in these extracts. These studies provide specific substrate and mechanistic information important for understanding the function of these aminopeptidases and could be exploited in the design of new inhibitors to specifically target these for anti-malaria treatment.

## Introduction

Malaria is one of the deadliest infectious diseases of humans in the world. It is endemic in tropical and subtropical regions, with about 500 million cases of malaria infections and 1.4–2.6 million deaths each year [1]. Four *Plasmodium* species commonly infect humans (*P. vivax*, *P. malariae*, *P. falciparum* and *P. ovale*) [2], [3]. Among them *P. falciparum* is of special interest because it is the most lethal and responsible for most deaths, particularly in pregnant women and children under the age of five. Drugs such as chloroquine and mefloquine have played major roles in the treatment of malaria in the past. However, the spread of drug resistant parasites has meant that treatment has become increasingly reliant on artemisinin-based combination therapies (ACTs) [4], [5]. Accordingly, there is a pressing need to develop new anti-malarial drugs targeting biochemical pathways critical for parasite survival and/or transmission.

Malarial parasites digest the infected host's hemoglobin to obtain free amino acids [6]. These amino acids are used to maintain osmotic pressure within infected red blood cells, for protein synthesis during parasite development and reproduction, and to set-up a concentration gradient by which rare or absent amino acids are transported into infected red blood cell from host serum [7], [8]. Two metallo-aminopeptidases M1 alanyl aminopeptidase (*Pf*M1AAP) and M17 leucine aminopeptidase (*Pf*M17LAP) expressed by *P. falciparum* may be responsible for the terminal steps of hemoglobin digestion [9], [10], [11]. It is proposed that these enzymes hydrolyze small peptides generated by the endoproteolytic digestion of hemoglobin within the parasite's digestive vacuole to generate a pool of free amino acids. Prevention of *Pf*M1AAP and *Pf*M17LAP activity by aminopeptidase-specific inhibitors, such as bestatin, block development of malaria parasites *in vitro* and *in vivo*, suggesting that these enzymes are attractive targets for the development of a new class of anti-malaria drugs [12], [13], [14]. Recently, Valmourougane et al. [15] used the bestatin scaffold to develop several derivatives and employed these to explore the active sites of the two malaria enzymes. Subsequently, Harbut et al. [16] synthesised additional bestatin-based inhibitors that exhibited specificity for *Pf*M1AAP and *Pf*M17LAP enzymes and showed that these can block malaria growth in culture, thus indicating that both enzymes represent targets for anti-malaria drug design.

**Figure 1:**
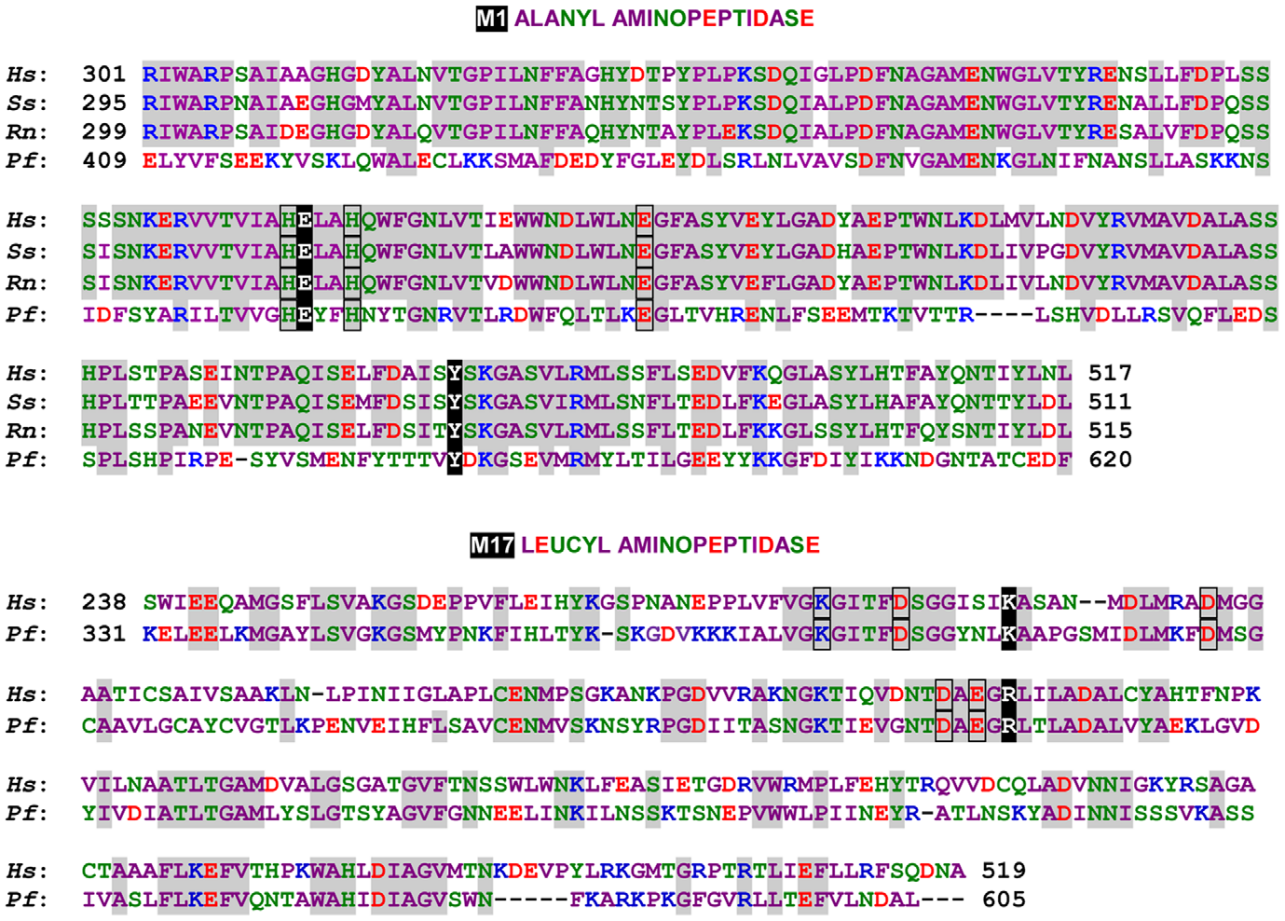
Multiple sequence alignments of *Pf*M1AAP and *Pf*M17LAP with mammalian orthologs, H: human *Homo sapiens*, P: pig, *Sus scrofa*, R: rat, *Rattus norvegicus* and X: *Plasmodium falciparum*. Dashes represent gaps that optimize sequence adjustment. Small or hydrophobic amino acids are colored in magenta, acidic are red, basic are blue and amino acids with an amine or hydroxyl group are green. Conserved amino acids are highlighted in gray. Amino acids from active side residues are presented on a black background and those participating in metal binding are outlined.

Sequence alignment of malaria *Pf*M1AAP and *Pf*M17LAP aminopeptidases with mammalian orthologs reveals significant differences in their overall primary structure and in residues that influence substrate binding (Figure 1). In particular, these data suggest that the S1 pockets of the malaria enzymes, which accepts the N-terminal P1 amino acids of a peptide substrate, has different topology in these enzyme orthologs, which could influence the binding and catalytic turnover of different classes of amino acids. Differences between the N-terminal amino acid preferences of malaria and mammalian enzymes could be exploited in the design of inhibitors that could kill malaria parasites without inhibition of their mammalian homologs.

In the present paper, we have examined and compared the detailed substrate specificities of functionally-active recombinant forms of *Pf*M1AAP and *Pf*M17LAP. To obtain substrate fingerprints for each enzyme we employed our recently developed substrate library consisting of natural and unnatural amino acids attached to an ACC fluorophore [17]. The information gained from this library provided extensive activity profiling of functional recombinant forms of *Pf*M1AAP and *Pf*M17LAP. We also profiled the general aminopeptidase activity in soluble cell lysates derived from the 3D7 clone of *P. falciparum* malaria. Our data show major differences in the substrate specificity between the two malaria enzymes that are related to the structure/shape of their active site. Significant difference was observed between these and the human aminopeptidase homologs for which we previously determined a substrate specificity profile [17]. Furthermore, we show that *Pf*M1AAP and *Pf*M17LAP represent the major aminopeptidase activity in soluble malaria extracts. This information will be important for the future characterization of the malaria aminopeptidases and elucidation of their function in malaria, and in the design of specific inhibitory compounds for anti-malarial treatment.

## Methods

### Materials

All chemicals and solvents were obtained from commercial suppliers and used without further purification, unless otherwise stated. Analytical high performance liquid chromatography (HPLC) analysis used a Beckman-Coulter System Gold 125 solvent delivery module equipped with a Beckman-Coulter System Gold 166 Detector system by using a Varian Microsorb-MV C18 (250×4.8 mm) column. Preparative HPLC analysis used a Beckman-Coulter System Gold 126P solvent delivery module equipped with a Beckman-Coulter System Gold 168 Detector system with a Kromasil 100-10 C18 (20 mm ID) column (Richard Scientific). Solvent composition system A (water/0.1%TFA) and system B (acetonitrile/water 80%/20% with 0.1% of TFA). LC-MS data were recorded with the aid of the Burnham Medicinal Chemistry facility using Shimadzu LCMS-2010EV system. The solid phase substrate library was synthesized using a semiautomatic FlexChem Peptide Synthesis System (Model 202). Enzymatic kinetic studies were performed using Spectra MAX Gemini EM fluorimeter (Molecular Devices) operating in the kinetic mode in 96-well plates.

### Preparation of malaria cell extracts and recombinant *Pf*M1AAP and *Pf*M17LAP

The intra-erythrocytic stages of 3D7 *P. falciparum* parasites were cultured in RPMI containing 10% human serum [18]. Parasites were lysed from erythrocytes using 0.03% saponin [19] and extracted by three rounds of freeze-thaw in phosphate-buffered saline, pH 7.3, prepared as described previously [20]. The production of recombinant malaria aminopeptidases *Pf*M1AAP and *Pf*M17LAP in *Escherichia coli* and their isolation by Ni-chelate affinity chromatography has been described elsewhere [21], [22].

### Substrate library screening

To first compare the substrate specificity of the two malarial aminopeptidases (*Pf*M1AAP and *Pf*M17LAP) an initial screening of 19 natural amino acids was performed. *Pf*M17LAP was assayed in 50 mM Tris-HCl, pH 8.0, and was activated with Co^2+^ ions at a final concentration of 1.0 mM. *Pf*M1AAP was assayed in 50 mM Tris-HCl, pH 7.5, without additional metal ions. These two buffers were made at 25°C and assays were carried out at 37°C. Before adding to the substrate, all enzymes were preincubated at 37°C for 30 minutes (the enzyme concentration in these additional screens was between 0.06–0.10 µM). These screens were carried out using substrate concentrations of 50 µM and 2.5 µM for *Pf*M1AAP and *Pf*M17LAP, respectively.

A final screening of the 61-membered library of the natural and unnatural amino acids was then performed: for *Pf*M1AAP screens the library concentration was 2.5 µM and for *Pf*M17LAP screens 300 nM (total enzyme concentrations in these assays were 0.03–0.10 µM). The fluorescence signal was monitored continuously and the wavelength values were following, excitation at 355 nm and emission at 460 nm. The total time of each assay was between 15–45 minutes. From each single experiment only the linear portion of progress curve was used to calculate final substrate specificity represented by RFU/s (production of Relative Fluorescence Unit per second) value. Experiments using the entire 61-membered library were repeated three times and for natural amino acids sublibrary were repeated twice. The average value with standard deviations for each substrate were compared with the best cleaved substrate (100% of specificity) and all data are presented on plot where x axis represents given fluorogenic substrate and y axis represents the specificity signified as percent participation in velocity of the most specific substrate.

### Determination of kinetic parameters for best cleaved substrates (K_m_, k_cat_, k_cat_/K_m_)

The kinetic parameters of the best substrates were determined using the above assay conditions. However, before adding to the substrate, all enzymes were preincubated at 37°C for 30 minutes. The ACC concentration was calculated by total digestion assay for each enzyme separately. In each measurement 6 independent substrates with known concentration were chosen and the average value was calculated. To measure K_m_ value eight different concentrations of given substrates and constant enzyme concentration were used. Reaction volume was at 100 µL and enzyme concentrations were 0.0315 µM and 0.380 µM for *Pf*M1AAP and *Pf*M17LAP, respectively. To measure k_cat_/K_m_ value six different concentrations of given substrates and constant enzyme concentration were used. All experimental conditions were as above. The fluorescence signal was monitored continuously and the wavelength value was the following, excitation at 355 nm and emission at 460 nm. The total time of each assay was between 15–30 minutes. All experiments were repeated at least three times and the average value with standard deviation was calculated. Concentration of DMSO in each experiment was less than 2% (v/v).

## Results

### Design of the substrate library

To determine substrate specificity of the enzyme-substrate complex in the S1 pocket of malaria aminopeptidases, we utilized a substrate-profiling approach in which a fluorogenic substrate library containing 61 amino acids was synthesized and used to profile three mammalian orthologs of the M1 aminopeptidase N [17]. This library was designed to screen substrate preferences for 19 natural amino acids (to avoid oxidation artifacts we omitted cysteine) and 42 unnatural amino acids representing a broad spectrum of side chain substitutions (Figure S1). Most of the compounds in the library contain an unblocked α-amino group to satisfy the primary specificity of aminopeptidases.

Additionally, we also synthesized several substrates with diverse functionalities (for example, a secondary amine derivatives, an α-hydroxy group, or an amine group in other than the α position) linked to a fluorophore leaving group (in the P1′ position) to determine how this would influence substrate recognition by malaria aminopeptidases. It was anticipated that this approach would provide additional information that could be used to identify good substrates, design of inhibitors, as well as comparison of different aminopeptidases. In our present investigation of substrate specificity, we used functionally-active recombinant forms of the two *P. falciparum* aminopeptidases - *Pf*M1AAP and *Pf*M17LAP (Figure 1) [9], [12]. As the fluorophore leaving group we employed 7-amino-4-carbamoylmethylcoumarin (ACC) because of its convenience in solid phase synthesis [23].

### Recombinant enzyme substrate-specificity results

An initial library screen for each malaria aminopeptidase was performed to establish optimal screening conditions. For each enzyme, the best cleaved substrates were chosen and their kinetic parameters (K_m_, k_cat_/K_m_, k_cat_) measured. After measurement of K_m_ we performed a second screen in which the concentration of each substrate was maintained well below the lowest K_m_ value. This procedure ensures that substrate cleavage (measured as a fluorescence signal) is proportional only to k_cat_/K_m_ and is not correlated with individual values of K_m_ or k_cat_. An equal concentration of the given substrates in each well was obtained by placing calculated amounts of substrate in the well and then mixing with enzyme to a total volume of 100 µL. Final substrate concentrations for the enzymes were as follows: 10 µM for *Pf*M1AAP (lowest K_m_ = 60.8 µM) and 0.3 µM for *Pf*M17LAP (lowest K_m_ = 0.35 µM). It is important to note that the most challenging library screening was with the *Pf*M17LAP. To obtain satisfactory fluorescence signals and avoid depletion of substrate at high enzyme concentration we performed the screen at 0.3 µM, only slightly below the K_m_ value of the best-cleaved substrate – Igl.

To gain a better insight into substrate specificity of the enzymes toward natural amino acids, we performed an additional screen at higher substrate concentration. This did not affect the observed data because the K_m_ values recorded for these substrates were also higher, which guaranteed a proportional correlation between fluorescence signal and k_cat_/K_m_: 50 µM for *Pf*M1AAP (lowest K_m_ = 138.2 µM) and 2.5 µM for *Pf*M17LAP (lowest K_m_ = 3.44 µM).

### *Pf*M1AAP aminopeptidase

**Figure 2:**
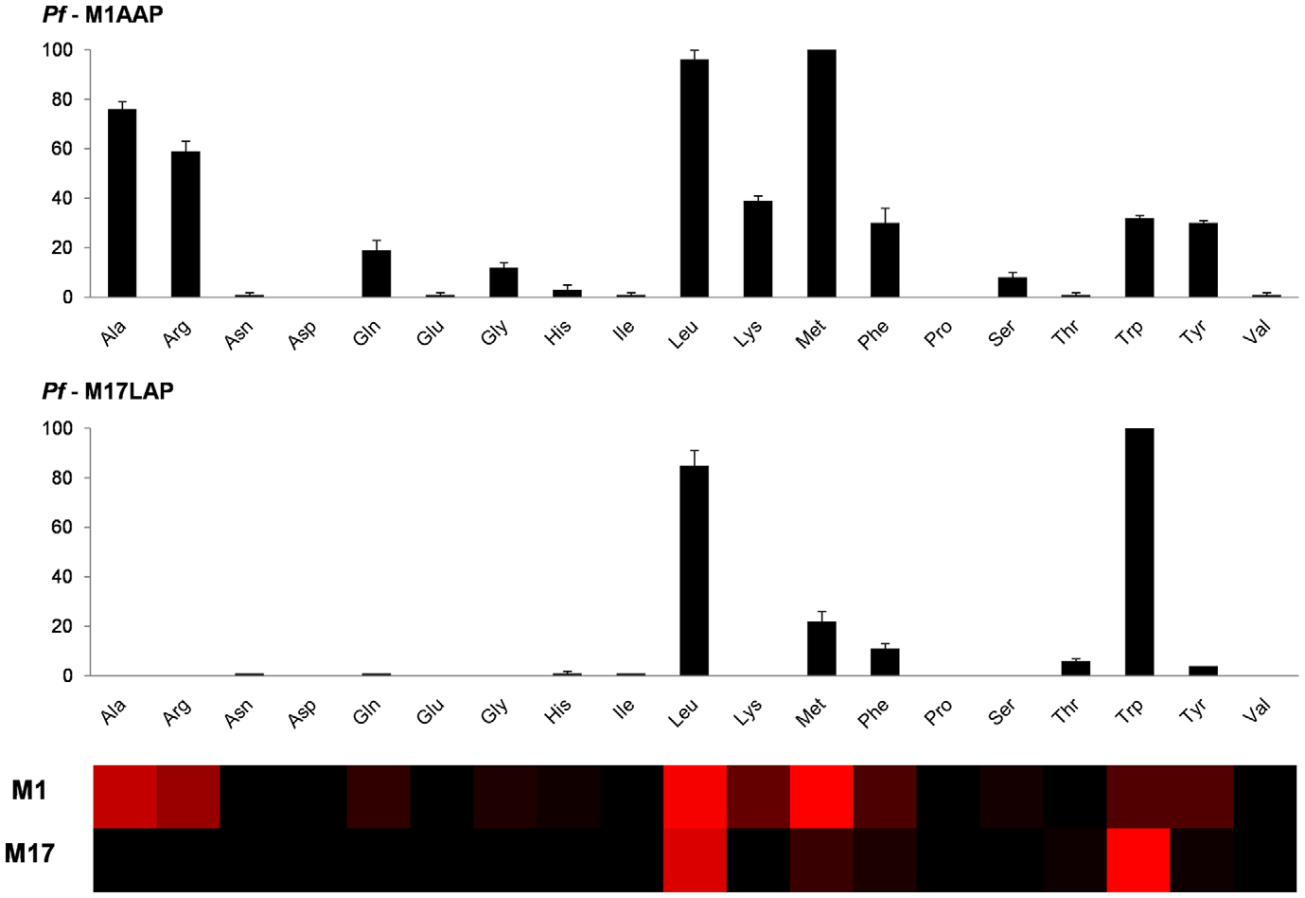
Preferred natural amino acids substrates for recombinant *Pf*M1AAP and *Pf*M17LAP. Initial screening of the 19-membered natural amino acid library. Enzyme activity was monitored using an fmax multi-well fluorescence plate reader (Molecular Devices) at excitation wavelength of 355 nm and an emission wavelength of 460 nm. he x-axis represents the abbreviated amino acid names (for full name and structure see Figure S1). The y-axis represents the average relative activity expressed as a percent of the best amino acid. In the heat map view the most preferred positions are displayed in bright red, whereas a complete lack of activity is in black, with intermediate values represented by intermediate shades of red.

The natural amino acids preferred by *Pf*M1AAP are leucine and methionine (Figure 2). Alanine and arginine are also readily cleaved by this enzyme, but with a slightly lower affinity. Other amino acids susceptible to hydrolysis by *Pf*M1AAP aminopeptidase include Lys, Phe, Tyr, Trp, Gln, Ser and Gly. Negligible activity was observed for Glu, Asp, Pro, Ile, Thr, Val, His and Asn.

**Figure 3:**
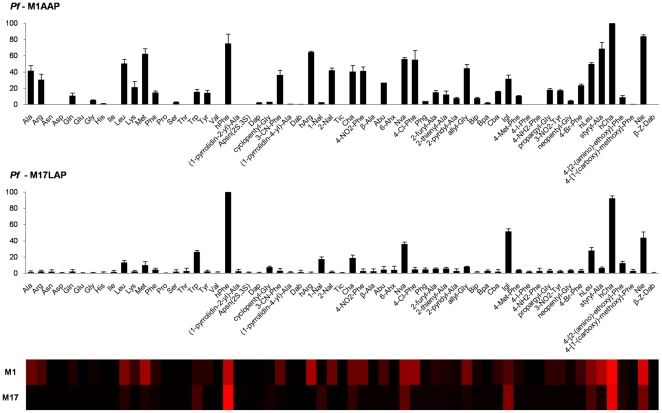
Individual preferences in S1 pocket of *Pf*M1AAP and *Pf*M17LAP enzymes toward natural and unnatural amino acid substrates. Screening of the 61-membered natural and unnatural amino acid library. Enzyme activity was monitored using an fmax multi-well fluorescence plate reader (Molecular Devices) at excitation wavelength of 355 nm and an emission wavelength of 460 nm. The x-axis represents the abbreviated amino acid names (for full name and structure see Figure S1). The y-axis represents the average relative activity expressed as a percent of the best amino acid. In the heat map view the most preferred positions are displayed in bright red, whereas a complete lack of activity is in black, with intermediate values represented by intermediate shades of red.

Analysis of the whole library revealed that *Pf*M1AAP exhibits very broad substrate specificity with this aminopeptidase capable of cleaving a range of substituents particularly the bulky, hydrophobic amino acids (Figure 3). The most preferred substrates were hCha, hPhe and Nle, all of which were cleaved more efficiently than the best natural amino acid, methionine. A second series of unnatural amino acids were also hydrolyzed by *Pf*M1AAP at about 50% of the activity seen for methionine. These were hArg, Cha, Nva, 4-Cl-Phe, 2-Nal, Igl, hLeu or styryl-Ala.

### *Pf*M17LAP aminopeptidase

*Pf*M17LAP exhibited strikingly narrow substrate specificity toward natural amino acids, particularly in comparison to *Pf*M1AAP (Figure 2). The most readily accepted substrates are the hydrophobic amino acids Leu and Trp. Other amino acids susceptible to hydrolysis, albeit at a very much lower level included Phe, Met, Thr and Tyr. Cleavage of Ile and His were slightly above background.

Analysis of the complete substrate library revealed a highly restricted specificity of *Pf*M17LAP for hydrophobic substrates (Figure 3). The amino acid derivatives most efficiently cleaved by *Pf*M17LAP were hPhe and hCha. These two substrates are cleaved approximately three and five times better than the best-cleaved natural amino acids Trp and Leu, respectively. Other amino acid derivatives that are cleaved by *Pf*M17LAP include Igl and Nle, and less so Nva and hLeu.

To study the distinct substrate differences between *Pf*M1AAP and *Pf*M17LAP in more detail we determined their kinetic parameters (K_m_, k_cat_, k_cat_/K_m_) against a panel of selected natural and unnatural substrates (Table 1). These studies showed that the substrates Arg, Ala, HArg, 2-Nal, 3-NO_2_-Phe and styryl-Ala were exclusively cleaved by *Pf*M1AAP. By contrast, we did not observe cleavage of any substrate by *Pf*M17LAP that was not cleaved by *Pf*M1AAP. For those substrates that both enzymes cleave, the efficiency or turnover rate (k_cat_/K_m_) was always far higher with *Pf*M1AAP in comparison to *Pf*M17LAP, even for those substrates most preferred by *Pf*M17LAP (e.g. Leu, hLeu, Phe, hPhe, hCha). However, the substrate binding affinities, as assessed by K_m_, for *Pf*M17LAP were between one or two orders of magnitude lower as compared to *Pf*M1AAP. These data indicate that the two enzymes function in milieu of different substrate concentration; *Pf*M1AAP works more effectively at relatively high substrate concentration while *Pf*M17LAP functions more efficiently at much lower substrate concentrations.

**Table 1:** Kinetic parameters for selected substrates.

	*Pf* M1AAP			*Pf* M17LAP		
	K_m_, µM	k_cat_, s^−1^	k_cat_/K_m_, M^−1^ s^−1^	K_m_, µM	k_cat_*10^3^, s^−1^	k_cat_/K_m_, M^−1^ s^−1^
Ala	240.6±10.9	1.054±0.154	4379±444	not cleaved		
Arg	214.3±18.5	0.876±0.105	4086±187	not cleaved		
hArg	124.3±15.6	1.144±0.100	9206±507	not cleaved		
Leu	140.9±12.1	0.868±0.111	6159±301	30.32±1.48	2.622±0.077	82.5±5.2
hLeu	210.8±26.6	1.859±0.030	8819±1481	4.059±0.017	0.717±0.121	176.6±15.5
Phe	218.0±6.2	0.422±0.028	1937±159	9.267±0.356	1.668±0.109	180.0±7.2
hPhe	60.8±3.1	0.978±0.003	16097±1754	0.595±0.004	0.277±0.029	466.3±51.3
Met	138.2±7.9	0.887±0.077	6420±200	3.440±0.297	0.117±0.007	34.1±1.8
Trp	144.4±18.7	0.444±0.052	3071±107	22.99±0.686	3.194±0.227	139.0±5.9
Cha	269.8±30.8	1.358±0.140	5034±692	7.777±0.520	0.839±0.125	107.9±13.3
hCha	96.3±16.2	1.592±0.230	16532±431	0.437±0.016	0.211±0.005	483.2±18.3
2-NaI	316.6±24.9	0.884±0.018	2792±248	not cleaved		
3-NO_2_-Phe	131.7±19.9	0.141±0.026	1072±221	not cleaved		
Nva	267.1±7.7	2.786±0.228	10429±1158	8.487±0.310	0.923±0.141	108.8±13
allyl-Gly	266.7±25.2	1.520±0.301	5700±720	11.35±1.89	0.479±0.106	42.3±4.3
IgI	84.7±6.1	0.492±0.032	5811±302	0.342±0.022	0.153±0.006	448.9±29
styryl-Ala	192.7±10.5	0.937±0.154	4864±556	not cleaved		
Nle	110.1±13.6	1.399±0.119	12708±1180	3.293±0.076	0.618±0.090	187.8±11.8

Comparison of the kinetic parameters (K_m_, k_cat_, k_cat_/K_m_) of the selected substrates for *Pf*M1AAP and *Pf*M17LAP. The results are presented as mean values with standard deviation.

### Malaria cell lysate substrate specificity results

To understand the nature of the aminopeptidase activity expressed by malaria parasites we screened our substrate library with a soluble malaria cell extract derived from the 3D7 clone of *P. falciparum*. We employed the substrate library at an arbitrary final concentration of 5 µM. This concentration was determined in a preliminary screening test to be sufficient to obtain a good and linear fluorescence signal (data not shown). No activity was observed against fluorogenic peptides substrates when lysates of uninfected erythrocytes were prepared in a similar manner to the parasite-infected erythrocytes as reported previously [9].

**Figure 4:**
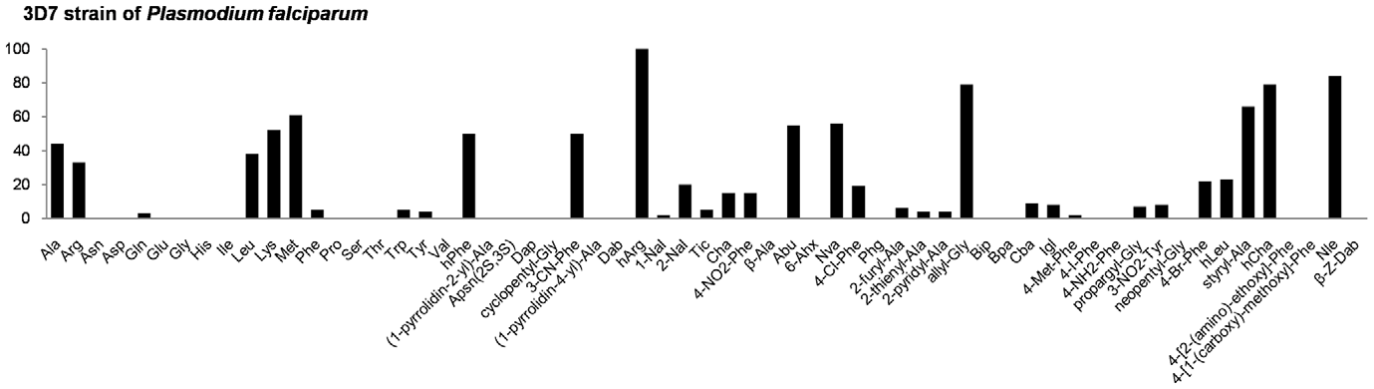
Total aminopeptidase activity explored in the 3D7 clone of *P. falciparum* in the absence (A). Aminopeptidase activity in soluble malaria parasite extracts was monitored using an fmax multi-well fluorescence plate reader (Molecular Devices) at excitation wavelength of 355 nm and an emission wavelength of 460 nm. The x-axis represents the abbreviated amino acid names (for full name and structure see Figure S1). The y-axis represents the average relative activity expressed as a percent of the best amino acid. Note, the addition of the aminopeptidease-specific inhibitor hPhe-PO_3_H_2_ (50 µM) to any of the above experiments completely abrogated cleavage of every substrate, thus confirming that the observed signal originates only from these enzymes.

Our data demonstrate that several substrates are efficiently cleaved by aminopeptidases in the cell lysate (Figure 4). Interestingly, the substrate profile closely represents a combination of activity of both *Pf*M1AAP and *Pf*M17LAP aminopeptidases. The most readily cleaved substrates (e.g. Arg, Ala, Leu, Met, hCha) show a close overlap with those cleaved by either recombinant enzymes. To validate this observation we performed a library screen in which the soluble cell lysate was preincubated for 30 minutes with 50 µM hPhe-PO_3_H_2_, which we have previously shown is a potent inhibitor of both *Pf*M1AAP and *Pf*M17LAP [14], [24]. No activity was observed toward any substrate in the complete library (data not shown). Thus we propose that the observed hydrolysis of the substrates by malaria soluble cell lysates results solely from the two aminopeptidases.

## Discussion

Malaria is currently considered one of the most deadly infectious global diseases of humans, killing approximately 1 million people in sub-Saharan Africa alone each year. New approaches to overcome the spread of malaria parasites that have become resistant to currently available drugs are necessary, particularly the identification of novel drugs targeting metabolic pathways. The aminopeptidases *Pf*M1AAP and *Pf*M17LAP are critical to the growth and development of malaria parasites within the erythrocyte as knockout of either aminopeptidase gene is lethal to the parasite [11], [12], and therefore they are both currently considered as promising targets for medicinal intervention [12]. The two enzymes are suggested to participate in the final step of hemoglobin digestion, the main source of nutrient for the parasite, resulting in the production of single amino acids, which are subsequently used for production of parasite proteins as they grow and develop with the host erythrocyte. As much as 70% of the erythrocyte hemoglobin is degraded suggesting that an efficient catabolic process is required [6]. However, the aminopeptidases may also function in the regular catabolic turnover of malaria proteins or biogenesis of intracellular organelles as the parasite undergoes recognized stage-specific developments [9], [10], [24].

Previous studies in the search for phosphonate or phosphinate compounds that inhibit both *Pf*M1AAP and *Pf*M17LAP resulted in the selection of several compounds that significantly reduced development of malaria parasites both in erythrocyte cell culture and in the murine *P. c. chabaudi* model of malaria [14], [25]. However, since these compounds block the activity of both enzymes it remains to be determined whether killing is due to inhibition of one or both enzymes. Harbut et al. [16] recently used a bestatin scaffold to develop inhibitors that showed a 12–15 fold specificity for either *Pf*M1AAP or *Pf*M17LAP and demonstrated that these killed malaria parasites *in vitro*. The *Pf*M1AAP-specific inhibitors caused swelling of the malaria digestive vacuole and disrupted proteolysis of haemoglobin-derived peptides while the *Pf*M17LAP-specific inhibitors killed malaria parasite prior to the onset of haemoglobin digestion. These support the idea that the two enzymes play distinct roles in malaria parasites and that both can be targeted for anti-malaria drug development [12].

Recently, the high-resolution X-ray crystal structures of both *Pf*M1AAP and *Pf*M17LAP were determined and revealed large differences within the S1 pockets of their active sites [19], [20]. Both molecules revealed extensively buried active sites centered around the essential active site cation(s). However the nature and size of the S1 pocket varied dramatically. The *Pf*M1AAP S1 pocket is long and contains acidic residues deep in the pocket, thus forming an excellent platform for docking amino acids of basic character. Notably, a polar glutamic acid (Glu^572^) residue is located at the base of the pocket where it would be available to form an ionic interaction with the side chains of long and basic side chains. Comparison of bestatin-bound and unbound *Pf*M1AAP structures also revealed flexibility of polar residues deep within the S1 pocket, thus possibly providing further adaptability to the shape of the S1 pocket. Valmourougane et al. [15] showed using bestatin-based inhibitors that the S1 pocket, residues 570–575, is flexible and can move to accommodate large side chains. Our library-screening results confirm that *Pf*M1AAP aminopeptidase can cleave a large variety of amino acids with small or bulky amino acids side chains. One of the best cleaved are compounds with Arg and hArg, thus confirming at a mechanistic level the crystal structure data analysis and predictions. In contrast, the *Pf*M17LAP S1 pocket that interacts with the substrate P1 residue is a small, narrow and substantially hydrophobic. Structural analysis suggested that only hydrophobic amino acids could be tolerated in this binding pocket. In the bestatin-bound structure, the P1 Phe-like moiety was tightly packed into the S1 pocket, forming stacking interactions with the hydrophobic pocket. Analysis of substrate library data for *Pf*M17LAP confirms predictions from its crystal structure by showing that this enzyme efficiently cleaves amino acids with bulky and hydrophobic side chains, while the presence of any hydrophilic group leads to reduced binding. The size and hydrophobic nature of this narrow pocket explains the inability of this enzyme to cleave peptides/proteins after polar residues. Analysis of the *Pf*M17LAP structure reveals no suitable polar hydrogen bonding partners at the base of the S1 pocket that could interact with a charged P1 side chain.

Substrates capable of differentiating between the two malaria aminopeptidases are Ala, Arg and hArg, a property that can be applied in the future for the specific monitoring activity of *Pf*M1AAP in cell lysates as well as for design of specific inhibitors for this enzyme. On the other hand, both *Pf*M1AAP and *Pf*M17LAP preferentially recognize and cleave two unnatural amino acids – hPhe and hCha. Phosphonate derivatives of these substrates were reported previously as very good inhibitors of recombinant *Pf*M17LAP and in malaria cell culture experiments, thus confirming that substrate specificity data can yield useful information for design of aminopeptidases inhibitors [14], [25].

Our previous studies using a restricted number of natural amino acid derivatives of fluorogenic substrates indicated that the two aminopeptidases exhibit distinct but overlapping substrate specificities [9], [19]. The availability of our library of 61 individual fluorogenic substrates in the form of natural and unnatural amino acids allowed us to perform comparative screens with the aminopeptidases, *Pf*M1AAP and *Pf*M17LAP. This has given us a more detailed understanding of the biochemistry of each *Pf*M1AAP and *Pf*M17LAP, which could enable the future design of specific substrates and inhibitors of each enzyme. The enzyme kinetic parameters presented in Table 1 show that the broad-acting *Pf*M1AAP cleaves all substrates that are also cleaved by the more restrictive *Pf*M17LAP. Moreover, *Pf*M1AAP cleaves these substrates with a far greater efficiency, with k_cat_/K_m_ values in the region of 40–100 fold higher. Additionally, the K_m_ values obtained for *Pf*M17LAP are between one or two orders of magnitude lower than those of *Pf*M1AAP. This further supports the suggestion that both enzymes may not function together in the same catabolic pathway and/or in the same cellular compartment. It is most probable that *Pf*M1AAP functions in a cellular environment where its substrates are in high concentration. Immunolocation studies [11], [21], [26], [27] suggest that this could possibly be within or adjacent to the parasites digestive vacuole where the initial endo- and exo-proteolytic cleavages of host hemoglobin would generate high concentrations of peptide substrates. In contrast, *Pf*M17LAP, which was localized to the cytoplasm of the malaria cell [9], [12], [21], could function where it substrates, peptides derived from hemoglobin or other proteins, are in lower concentration. Because of its strict specificity for leucine, we have previously suggested that a prime function of *Pf*M17LAP could be in generating high intracellular concentrations of leucine that can be exchanged via specific channels for extracellular isoleucine [28], an essential amino acid not found in human hemoglobin [9], [12].

One of the objectives of this study was to characterize the aminopeptidase activity of aminopeptidases in malaria extracts and compare this to the recombinant *Pf*M1AAP and *Pf*M17LAP. Our data clearly show that *Pf*M1AAP and *Pf*M17LAP are primarily responsible for the aminopeptidase activity in the soluble lysates of the 3D7 clone of *P. falciparum*. There are four types of methionine aminopeptidases (MetAP) expressed in malaria cells [29] and we expected that this activity would be particularly enhanced in the substrate profile of soluble cell lysates compared to the recombinant *Pf*M1AAP and *Pf*M17LAP. Interestingly, this was not the case and it is most probable that MetAP activities are presence at a low level in the soluble lysates, although other possibilities include that these enzymes are membrane bound. Our data strongly suggests that both *Pf*M1AAP and *Pf*M17LAP are the predominant exo-aminopeptidases in the soluble lysates of the malaria parasites.

In conclusion, we have used a new library of fluorogenic substrates designed from natural and unnatural amino acids to define the distinct substrate specificity and kinetic parameters of two malaria aminopeptidases *Pf*M1AAP and *Pf*M17LAP, potential targets for new anti-malarials. Aminopeptidase fingerprint of *Pf*M1AAP overlaps very well with previously published data for three mammalian (human, rat and pig) orthologs of this enzyme suggesting that no dramatic evolutionary changes occurred to this enzyme in term of substrate recognition preferences. This suggests that designing inhibitors that block the activity of the malaria enzyme without inhibiting the host enzyme will present a major challenge. However, our results show individual features of each malaria aminopeptidase in term of binding substrates in S1 pocket and suggest that compounds that inhibit each enzyme specifically or together could be synthesized for combination therapies. This suggestion is supported by the recent results of Valmourougane et al. [15] and Harbut et al. [16] who designed *Pf*M1AAP- and *Pf*M17LAP-specific inhibitors using the basic bestatin scaffold, although these did not show enhanced killing of parasite over bestatin itself. We have also shown that our library could be employed for activity profiling of cell extracts from different strains of malaria. Finally, our analysis can form the basis for future selection of specific substrates for this group of proteases as well as for the design of inhibitors, which could further help to answer questions about their relative importance in malaria development.

## Supporting Information

Figure S1 **Structures of natural and unnatural amino acids fluorogenic substrates used in the library.**
(DOC)
