# Supplementary material for: Fingerprinting the Substrate Specificity of M1 and M17 Aminopeptidases of Human Malaria, Plasmodium falciparum
Source: PLoS One. 2012 Feb 16;7(2):e31938. doi: 10.1371/journal.pone.0031938 (PMC3281095; doi:10.1371/journal.pone.0031938)
Supplement: Figure S1 — Structures of natural and unnatural amino acids fluorogenic substrates used in the library. (DOC) [file pone.0031938.s001.doc]

**Figure S1. Structures of natural and unnatural amino acids fluorogenic substrates used in the library.**

| **Entry** | **Structure** | **Name** | **M.W.** |
| --- | --- | --- | --- |
| 1 |  | Ala | 289.1 |
| 2 |  | Arg | 376.2 |
| 3 |  | Asn | 332.1 |
| 4 |  | Asp | 333.1 |
| 5 |  | Gln | 346.2 |
| 6 |  | Glu | 347.1 |
| 7 |  | Gly | 275.1 |
| 8 |  | His | 355.2 |
| 9 |  | Ile | 331.2 |
| 10 |  | Leu | 331.2 |
| 11 |  | Lys | 346.2 |
| 12 |  | Met | 349.1 |
| 13 |  | Phe | 365.2 |
| 14 |  | Pro | 315.1 |
| 15 |  | Ser | 305.1 |
| 16 |  | Thr | 319.1 |
| 17 |  | Trp | 404.2 |
| 18 |  | Tyr | 381.2 |
| 19 |  | Val | 317.2 |
| 20 |  | hPhe | 379.2 |
| 21 |  | (1-pyrrolidin  -2-yl)-Ala | 358.2 |
| 22 |  | Apns (2S,3S) | 395.2 |
| 23 |  | Dap | 304.1 |
| 24 |  | cyclopentyl-Gly | 343.2 |
| 25 |  | 3-CN-Phe | 390.2 |
| 26 |  | (1- piperidin-4-yl)-Ala | 372.2 |
| 27 |  | Dab | 318.2 |
| 28 |  | hArg | 388.2 |
| 29 |  | 1-Nal | 415.2 |
| 30 |  | 2-Nal | 415.2 |
| 31 |  | Tic | 377.2 |
| 32 |  | Cha | 371.2 |
| 33 |  | 4-NO_2_-Phe | 410.1 |
| 34 |  | β-Ala | 289.1 |
| 35 |  | Abu | 303.1 |
| 36 |  | 6-Ahx | 331.2 |
| 37 |  | Nva | 317.2 |
| 38 |  | 4-Cl-Phe | 399.1 |
| 39 |  | Phg | 351.1 |
| 40 |  | 2-furyl-Ala | 355.1 |
| 41 |  | 2-thienyl-Ala | 371.1 |
| 42 |  | 2-pyridyl-Ala | 366.1 |
| 43 |  | allyl-Gly | 315.1 |
| 44 |  | Bip | 441.2 |
| 45 |  | Bpa | 469.2 |
| 46 |  | Cba | 328.1 |
| 47 |  | Igl | 391.2 |
| 48 |  | 4-Met-Phe | 379.2 |
| 49 |  | 4-I-Phe | 491.0 |
| 50 |  | 4-NH_2_-Phe | 380.1 |
| 51 |  | propargyl-Gly | 313.1 |
| 52 |  | 3-NO_2_-Tyr | 426.1 |
| 53 |  | neopentyl-Gly | 345.2 |
| 54 |  | 4-Br-Phe | 443.1 |
| 55 |  | hLeu | 345.2 |
| 56 |  | styryl-Ala | 391.2 |
| 57 |  | hCha | 385.2 |
| 58 |  | 4-[2-(amino)-ethoxy]-Phe | 424.2 |
| 59 |  | 4-[1-(carboxy)-methoxy]-Phe | 439.2 |
| 60 |  | Nle | 331.2 |
| 61 |  | β-Z-Dab | 452.2 |
|  |  |  |  |
